# Ultra-high critical current densities of superconducting YBa_2_Cu_3_O_7-δ_ thin films in the overdoped state

**DOI:** 10.1038/s41598-021-87639-4

**Published:** 2021-04-14

**Authors:** A. Stangl, A. Palau, G. Deutscher, X. Obradors, T. Puig

**Affiliations:** 1 Institut de Ciència de Materials de Barcelona (ICMAB-CSIC) Campus de Bellaterra, 08193 Bellaterra, Barcelona, Spain; 2 Department of Physics and Astronomy, Tel Aviv University, 69978 Tel Aviv, Israel

**Keywords:** Materials science, Condensed-matter physics, Superconducting properties and materials

## Abstract

The functional properties of cuprates are strongly determined by the doping state and carrier density. We present an oxygen doping study of YBa_2_Cu_3_O_7-δ_ (YBCO) thin films from underdoped to overdoped state, correlating the measured charge carrier density, ${n}_{\mathrm{H}}$, the hole doping, *p*, and the critical current density, ${J}_{c}$. Our results show experimental demonstration of strong increase of ${J}_{c}$ with ${n}_{\mathrm{H}}$, up to Quantum Critical Point (QCP), due to an increase of the superconducting condensation energy. The ultra-high ${J}_{c}$ achieved, 90 MA cm^−2^ at 5 K corresponds to about a fifth of the depairing current, i.e. a value among the highest ever reported in YBCO films. The overdoped regime is confirmed by a sudden increase of ${n}_{\mathrm{H}}$, associated to the reconstruction of the Fermi-surface at the QCP. Overdoping YBCO opens a promising route to extend the current carrying capabilities of rare-earth barium copper oxide (REBCO) coated conductors for applications.

## Introduction

High temperature superconductors (HTS) are key materials for many applications, with special interest in those related to the energy sector^1,2^. However, to reach these opportunities, outstanding research had to be made within the last 30 years from the discovery of high temperature superconductivity, to overcome the most critical materials issues^3,4^. The successful development of superconducting Coated Conductors (CC) is based on the uniform textured deposition of thick, homogeneous nanoengineered structures in kilometres length (a desired correlation over twelve orders of magnitude!). Cuprate materials in the form of CCs have opened up new opportunities in the field of superconducting applications^5^ and nowadays offer the performance to boost technological innovations such as dissipation-free energy transmission in superconducting grids, highly efficient engines for electrical aviation or compact fusion reactors beyond ITER. However, current carrying capacities are still far from theoretical limits^6,7^.

While on one hand dissipation free current transport is intrinsically limited by the depairing current density, ${J}_{\mathrm{d}}$, beyond which Cooper pair condensation is energetically not favourable, in real systems the dissipation free, critical current density, ${J}_{c}$ is smaller due to the motion of magnetic vortices. One major goal in the development of superconducting tapes is to merge ${J}_{c}$ with the theoretical limit, ${J}_{\mathrm{d}}$. Nowadays, the main approach to increase ${J}_{c}$ is the introduction of nanoscaled defects (artificial pinning centres) in the YBCO matrix to immobilize magnetic vortices^8–13^. The total pinning force, ${F}_{\mathrm{p}}={J}_{c}\times B$, is enhanced by an increased number of elementary pinning sites of optimised size and distribution^14^. However, the normal and superconducting (SC) state properties of cuprate materials are strongly governed by hole doping of the superconducting CuO_2_-planes. Likewise, the elementary pinning strength itself varies with the condensation energy, ${E}_{c}$, per coherence volume which was predicted to peak at the critical doping p* = 0.19 where the pseudogap closes^15^. In^16^ it was reported that the mean field value of the heat capacity jump at the critical temperature, ${T}_{\mathrm{c}}$, and therefore the condensation energy, increases up to the maximum oxygen stoichiometry O_7_, beyond optimal doping, in bulk YBCO. More recently, a strong increase of ${E}_{c}$, between the optimal doping (*p* = 0.16) and p* was found by measurements of the critical fields^17,18^. These results further emphasize the enhancement of ${J}_{c}$ by overdoping YBCO (as confirmed by results of the doping level), which motivated the interest of early studies to achieve overdoped YBCO films^19–23^. Nevertheless, demonstration of overdoping of YBCO films by oxygen doping has been quite scare up to now.

In this work, we have reached the overdoped state by oxygen post-processing of YBCO thin films with a thickness of 200 and 250 nm, grown from pulsed laser deposition (PLD) and chemical solution deposition (CSD), respectively. We show the influence of oxygen doping on the charge carrier concentration determined by Hall effect measurements all the way to the overdoped state. We find that ${J}_{\mathrm{c}}$ strongly increases with charge carrier density, far into the overdoped state where a large Fermi surface of well-defined quasiparticles exists^24^. We demonstrate that overdoped YBCO films achieve record ${J}_{\mathrm{c}}$ values of 90 MA/cm^2^ at 5 K self-field, reaching a fifth of the depairing current density. Our results are in line with recent proposals of the controversial extrapolation of the pseudogap line, its relationship with the Quantum Critical Point and a Fermi Surface reconstruction at the critical point^25–27^, which are crucial issues to understand the nature of high-T_c_ superconducting cuprates, particularly the overdoped state. We foresee the hybridization of overdoping and nanoengineering of HTS-CC as an emerging opportunity to significantly improve CC performances for a broad range of superconducting energy applications.

## Results

### Normal state electrical properties and doping state of YBCO thin films

**Figure 1:**
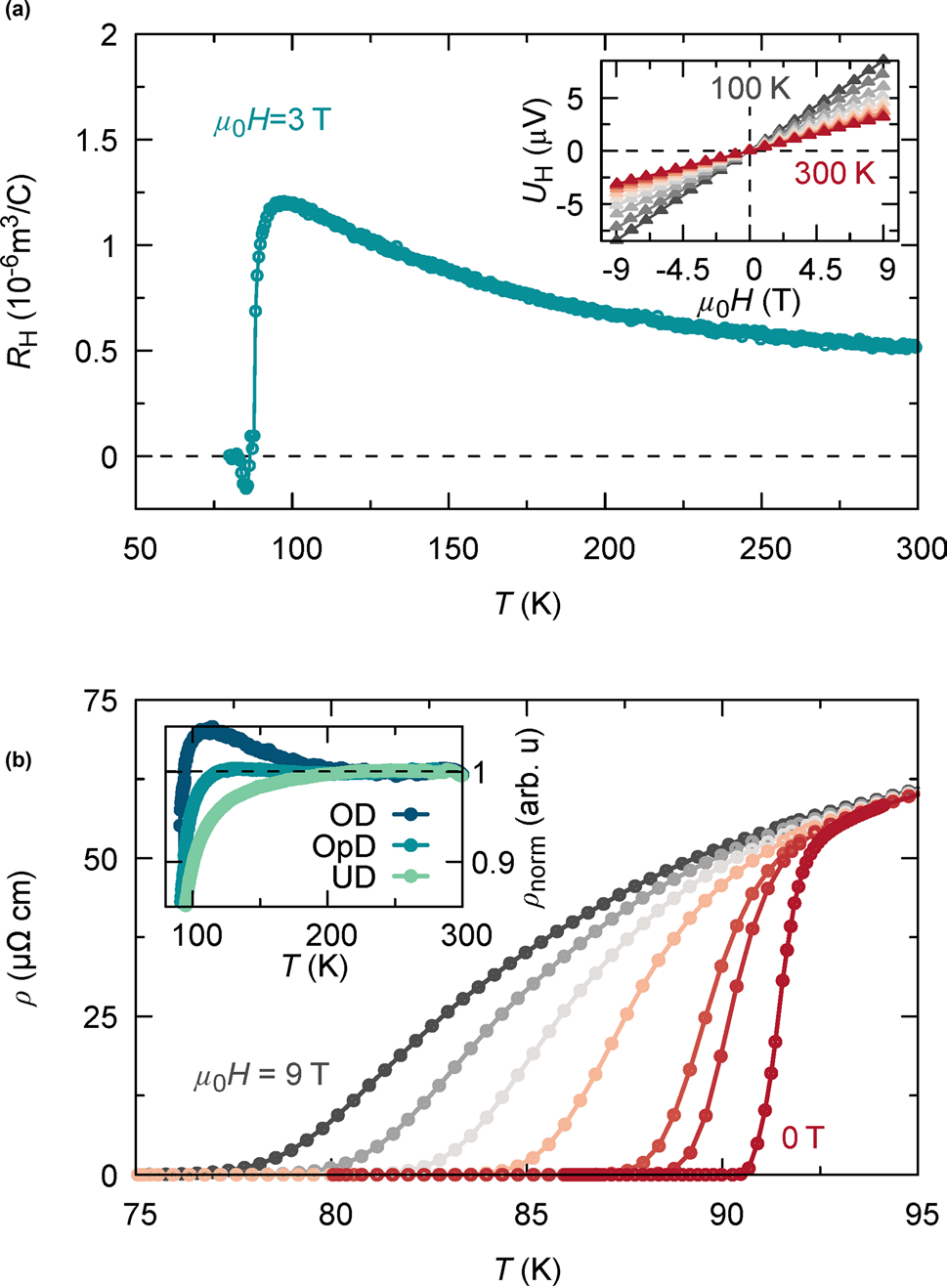
Electrical analysis of YBCO thin films. (**a**) Hall constant ${R}_{\mathrm{H}}$ obtained at 3 T as a function of temperature. ${R}_{\mathrm{H}}$ is perfectly linear within the analysed field range (− 9 to 9 T) and vanishes in zero field condition at all *T* as shown in the inset. Charge carrier density is obtained via Hall effect measurements using ${n}_{\mathrm{H}}(T)=\frac{1}{{R}_{\mathrm{H}}(T)q}$. (**b**) In-plane resistivity, $\rho (T,H)$, as a function of temperature at different magnetic fields (0, 0.5, 1, 3, 5, 7, 9 T, $H\parallel c)$ to evaluate the field dependent superconducting transition temperature, ${T}_{0}(H)$. Inset shows normalized resistivity, ${\rho }_{\mathrm{norm}}=(\rho \left(T\right)-{\rho }_{0})/bT$, where $b$ is the linear slope at high temperatures ($T>150\hspace{0.17em}\mathrm{K})$. Doping dependent deviation from unity is observed, as shown for an underdoped (UD, downwards deviation), optimally doped (OpD) and overdoped (OD, upwards bending) 200 nm thick YBCO film, respectively.

**Figure 2:**
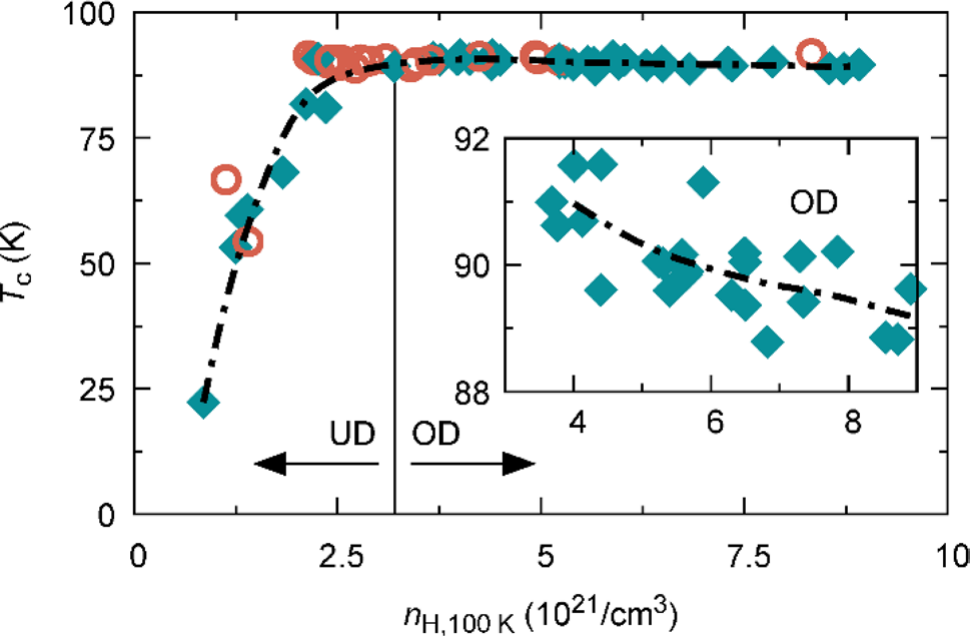
Phase diagram of YBCO thin films. Zero field critical temperature as a function of charge carrier density, ${n}_{H}$, obtained by Hall effect measurements at 100 K for thin YBCO films grown by PLD (200 nm thick, cyan diamonds) and CSD (250 nm thick, red circles). Vertical line marks optimally doping, while arrows indicate underdoped (UD) and overdoped (OD) regime. Inset magnifies ${T}_{c}$ in the overdoped regime, showing a weak but distinct decrease with increasing charge carrier density, from above 91 K to around 89 K.

Normal state electrical properties of cuprate superconductors strongly vary with doping. Underdoped YBCO thin films were obtained by using oxygen partial pressure below 1 bar during the post growth oxygenation process. Overdoped YBCO films were achieved by post-growth oxygen heat treatments at low temperatures, enabled by catalytically activated surface oxygen exchange, using a thin Ag surface decoration layer (for details see Supplementary Information SI-Fig. 1). The catalytic effect occurs via a job sharing mechanism where Ag dissociates the molecular oxygen and YBCO incorporates the oxygen ion into the bulk^28^. Electrical resistance and Hall measurements as a function of temperature have been employed in addition to room temperature *c*-parameter analysis to determine the doping state of the studied films. Representative examples of the temperature dependence of the Hall resistance ${R}_{\mathrm{H}}(T)$ and the electrical resistivity ${\rho}(T,H)$ at different magnetic fields are given in Fig. 1 for our films. We have inferred the charge carrier density ${n}_{\mathrm{H}}(T)=\frac{1}{e{R}_{\mathrm{H}}(T)}$ by Hall measurements at constant magnetic field (3 T) and sweeping temperature (SI-Fig. 2). We observe a strong temperature dependence of ${R}_{\mathrm{H}}$, respectively ${n}_{\mathrm{H}}$, as previously reported for HTS cuprates^29,30^, with a maximum of ${R}_{\mathrm{H}}$ around 100 K as shown in Fig. 1a. A similar drop of ${R}_{\mathrm{H}}(T)$ below 100 K in underdoped YBCO was recently explained by a reconstruction of the Fermi surface in the normal state due to charge-density-wave order^31,32^. However, in the present case the downturn in ${R}_{\mathrm{H}}$, which is also observed in optimally and overdoped YBCO, is caused by the onset of superconductivity, as the applied magnetic field ($\mu {H}_{\mathrm{a}}=3$ T) is too weak to suppress the occurring phase transition to the superconducting state. In this work, we use the charge carrier density extracted at 100 K to study the correlation with superconducting properties, such as the critical current density.

The resistivity, $\rho (T,H)$ in Fig. 1b reveals a broadening of the superconducting transition width with increasing magnetic field. From $\rho (T,H)$ measurements we have inferred the coherence length, $\xi (0)$, and a characteristic magnetic field, ${H}_{0}$, as discussed below. The deviation of $\rho \left(T\right)$ from a linear temperature dependence at low *T* is shown in the inset of Fig. 1b which correlates with under- and overdoping, respectively, consistent with previous reports^33^.

Figure 2 displays the critical temperature as a function of the Hall number, ${n}_{\mathrm{H}}(100\hspace{0.17em}\mathrm{K})$, measured at 100 K. ${T}_{\mathrm{c}}$ rapidly increases at low values of ${n}_{\mathrm{H}}$, as expected from the parabolic dependence of ${T}_{c}$ on doping generally found in cuprate superconductors^34^ (assuming direct proportionality between the charge carrier density and doping in the underdoped region). Above the optimal doping of about ${n}_{\mathrm{H}}(100\hspace{0.17em}\mathrm{K})=3\cdot {10}^{21}$/cm^3^, ${T}_{\mathrm{c}}$ decreases, but following a much weaker dependence, as shown in the inset. This deviation from a parabolic dependence of ${T}_{\mathrm{c}}$ was reported previously^33^ and can be explained by an analysis of the doping dependence of the charge carrier density, as performed in the following.

**Figure 3:**
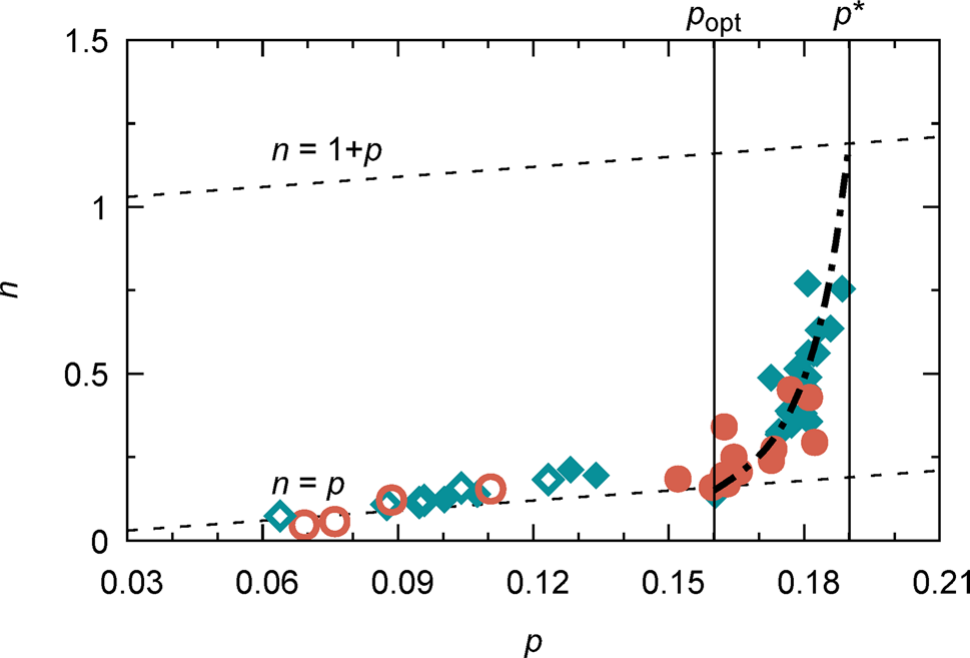
Evolution of the charge carrier density with doping. YBCO normal state charge carrier density per CuO_2_-plane, *n*, is drawn as a function of doping $p$. The charge carrier density is given by $n=\frac{{n}_{\mathrm{H}}V}{2}$ at 100 K. The doping $p$ is obtained for optimally and overdoped samples (full symbols) via HR-XRD measurements of the $c$-parameter and for underdoped films (open symbols) from the parabolic doping dependence of ${T}_{c}$. The vertical lines indicate optimally and critical doping. Below ${p}_{\mathrm{opt}}$ we find $\mathrm{n}=\mathrm{p}$, corresponding to a Fermi-surface with small hole and/or electron pockets in the underdoped regime. For $p>{p}^{*}$ a large, cylindrical FS is expected in the metallic overdoped regime, with $\mathrm{n}=1+\mathrm{p}$. A transition between a small and a large Fermi-surface occurs above $p=0.16$. This is in good agreement with previous reports, but remarkable, as within this work $\mathrm{n}$ is obtained by Hall effect measurements using small fields above the onset of superconductivity at 100 K.

In the underdoped regime, we calculated $p$ from measurements of ${T}_{c}$ using the parabolic dependence $1-\frac{{T}_{\mathrm{c}}}{{T}_{\mathrm{c},\mathrm{max}}}=82.6{\left(p-0.16\right)}^{2},$ with ${T}_{\mathrm{c},\mathrm{max}}=92$ K, generally found for cuprates. However, this method was found insufficient for optimally and overdoped thin films. Hence, for these higher doped films we determined the doping number from measurements of the $c$-parameter by HR-XRD, as described in the methods (see also SI-Fig. 3a,b).

In Fig. 3 we plot the evolution of the charge carrier density, $n$, at *T* = 100 K with doping, $p$. Here we use the charge carrier density per Cu in the CuO_2_-planes, $n=\frac{{n}_{\mathrm{H}}V}{2}$, with the volume of the unit cell $V$ (the factor ½ is owing to the fact that YBCO has two CuO_2_-planes per unit cell). In this calculation, we assumed a constant $V$ = 0.173 nm^3^, owing to the fact that in the range of oxygen doping explored, the variation in $V$ is rather small (< 1%)^35^ while large variations in ${n}_{\mathrm{H}}$ occur. We notice that although the same general behaviour is observed for both types of films, both reaching the overdoped state, PLD films could usually reach a higher oxygenation state than CSD. This could probably be associated to different oxygenation kinetics affecting the microstructure of the film (strain, defects, etc.). For *p* < 0.16, we find $n=p$ in a broad doping range (underdoped regime). Above optimal doping (*p* > 0.16), $n$ sharply increases. A similar result was reported previously, where ${n}_{\mathrm{H}}$ was measured at low temperatures using very high magnetic fields suppressing the superconducting state (e.g. at 50 K and up to 88 T)^31^. This can be understood by a Fermi surface reconstruction (FSR) in proximity to the pseudogap (PG) critical point at p*, resulting in a non-unique relation between the charge carrier density, $n$, and doping, $p$, over the full range of the cuprate phase diagram^27^. In the far overdoped regime, cuprate superconductors have been recently shown to exhibit a large cylindrical Fermi Surface (FS) with $n(T\to 0)=1+p$ in the zero temperature limit^31,32,36,37^. Below the critical doping, the volume of the FS reduces by one hole per Cu in the CuO_2_ plane to $n(T\to 0)=p$, e.g. due to the introduction of an antinodal gap opening which seems to arise from short range antiferromagnetic correlations in the system^25^. This transition is expected to occur within a narrow doping range between the optimal doping $p=0.16$ and the closing of the PG at the critical doping ${p}^{*}=0.19$, as observed in our study. The PG critical point at p* has been shown to have all the features of a quantum critical point at $T=0$^38^.

It is remarkable that the charge carrier density measured at 100 K, above the onset of superconductivity, preserves the expected behaviour for $n\left(T\to 0\right)$ of a single band metal with a Fermi-surface containing small hole-like pockets on the underdoped site and a sharp transition towards a large Fermi-volume, due to a reconstruction of the Fermi surface, in overdoped YBCO. The latter is also the reason for the observed deviation from a parabolic doping dependence of ${T}_{c}$ on ${n}_{\mathrm{H}}$, shown in Fig. 2.

### Superconducting properties of YBCO films: Correlation with doping and condensation energy

**Figure 4:**
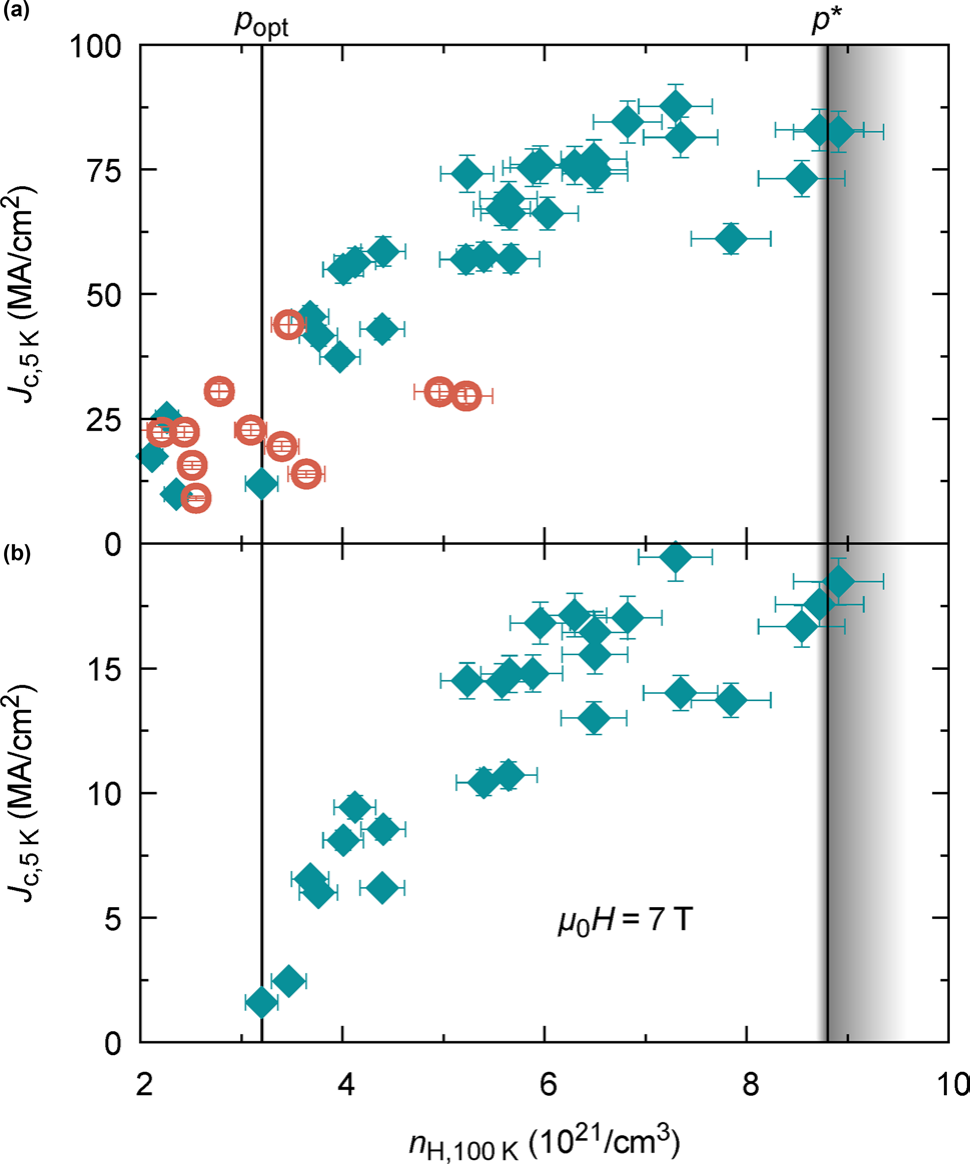
Dependence of ${J}_{c}$ on charge carrier density: Self-field inductive critical current density, ${J}_{c}$, at 5 K versus charge carrier density ${n}_{\mathrm{H}}(100\hspace{0.17em}\mathrm{K})$ in (**a**) self-field and (**b**) an applied magnetic field of 7 T of YBCO thin films obtained by CSD (red circles, 250 nm) and PLD (cyan diamonds, 200 nm). The critical current density is determined by SQUID magnetisation measurements. ${J}_{c}$ is strongly enhanced by increasing the charge carrier density far into the overdoped regime. Optimal and critical doping, ${p}_{opt}$ and ${p}^{*}$, are marked with vertical lines, while the shadowed area around ${p}^{*}$ indicates the uncertainty of defining the critical doping in terms of ${n}_{\mathrm{H}}$ via Fig. 3. Error bars correspond to uncertainties in film thickness.

The main result of this work is shown in Fig. 4, where we plot the inductive critical current density, ${J}_{\mathrm{c}}$, as a function of ${n}_{\mathrm{H}}(100\hspace{0.17em}\mathrm{K})$ in (a) at self-field and (b) at an applied magnetic field of 7 T, determined by magnetisation measurements at 5 K (SI-Fig. 4). For both cases, we find a strong increase of ${J}_{c}$ with ${n}_{\mathrm{H}}$, extending beyond optimal doping, far into the overdoped regime up to the equivalent value of p* = 0.19 (corresponding ${n}_{\mathrm{H}}$ values for optimal and critical doping are indicated with vertical dashed lines). Notice the ultrahigh values of inductive ${J}_{c}$ at self-field and 5 K, ${J}_{c}(5\hspace{0.17em}\mathrm{K})$, achieved beyond optimal doping up to p*.

An evaluation of the depairing current within the Ginzburg–Landau theory^39^ is done in the following in order to compare the obtained experimental values with this theoretical limit, though strictly valid only near ${T}_{\mathrm{c}}$:

1 $${J}_{\mathrm{d}}^{\mathrm{GL}}(T)=\frac{{\phi }_{0}}{{3}^{3/2}\pi {\mu }_{0}{\lambda }^{2}\left(T\right)\xi \left(T\right)}$$

with the temperature and material dependent magnetic penetration depth $\lambda \left(T\right)=\lambda \left(0\right)/ \sqrt{1-{t}^{4}}$ and coherence length $\xi \left(T\right)= \xi \left(0\right)\sqrt{(1+{t}^{2})/ (1-{t}^{2})}$, the flux quantum ${\phi }_{0}$ and the reduced temperature $t=T/{T}_{c}$. Using values for $\lambda \left(0\right)$ as reported in^40^ for YBCO bulk and $\xi \left(0\right)$ as obtained within this work, we can estimate ${J}_{\mathrm{d}}^{\mathrm{GL}}\left(5\hspace{0.17em}\mathrm{K}\right)$ for optimally and overdoped YBCO, as shown in Table 1. The ratio ${J}_{\mathrm{c}}/{J}_{\mathrm{d}}$ for YBCO thin films analysed within this work, approximately doubles from ~ 9% to ~ 18% going from optimally to overdoped (*p* = p*), and further evidences the relevance of going to the overdoped state. The maximum ${J}_{\mathrm{c}}(5\hspace{0.17em}\mathrm{K})$ obtained within this study is 89.4 MA/cm^2^, reaching about a fifth of the fundamental limit. To the best of the authors knowledge, this is among the highest inductive ${J}_{c}(5\hspace{0.17em}\mathrm{K})$ values reported for REBCO thin film superconductors at zero field^41–45^. We suggest, that this increase of ${J}_{c}$ in YBCO films with the charge carrier density up to p* is a consequence of the increase of the condensation energy with charge carrier density, as shown in the following.

**Table 1:** Estimation of the ratio of critical current density of this work, ${J}_{\mathrm{c}}$, over depairing critical current density, ${J}_{\mathrm{d}}$. $\xi (0)$ and ${J}_{\mathrm{c}}$ obtained within this work, $\lambda (0)$ from^40^ and ${J}_{\mathrm{d}}$ calculated using Eq. (1).

	ξ(0) (nm)	λ(0) (nm)	${J}_{\mathrm{c}}\left(5\hspace{0.17em}\mathrm{K}\right)$(MA/cm^2^)	${J}_{\mathrm{d}}(5\hspace{0.17em}\mathrm{K})$(MA/cm^2^)	${J}_{\mathrm{c}}/{J}_{\mathrm{d}}(5\hspace{0.17em}\mathrm{K})$
Optimal doped	1.7	134	30	330	9%
Overdoped (*p* = p*)	1.6	112	90	500	18%

**Figure 5:**
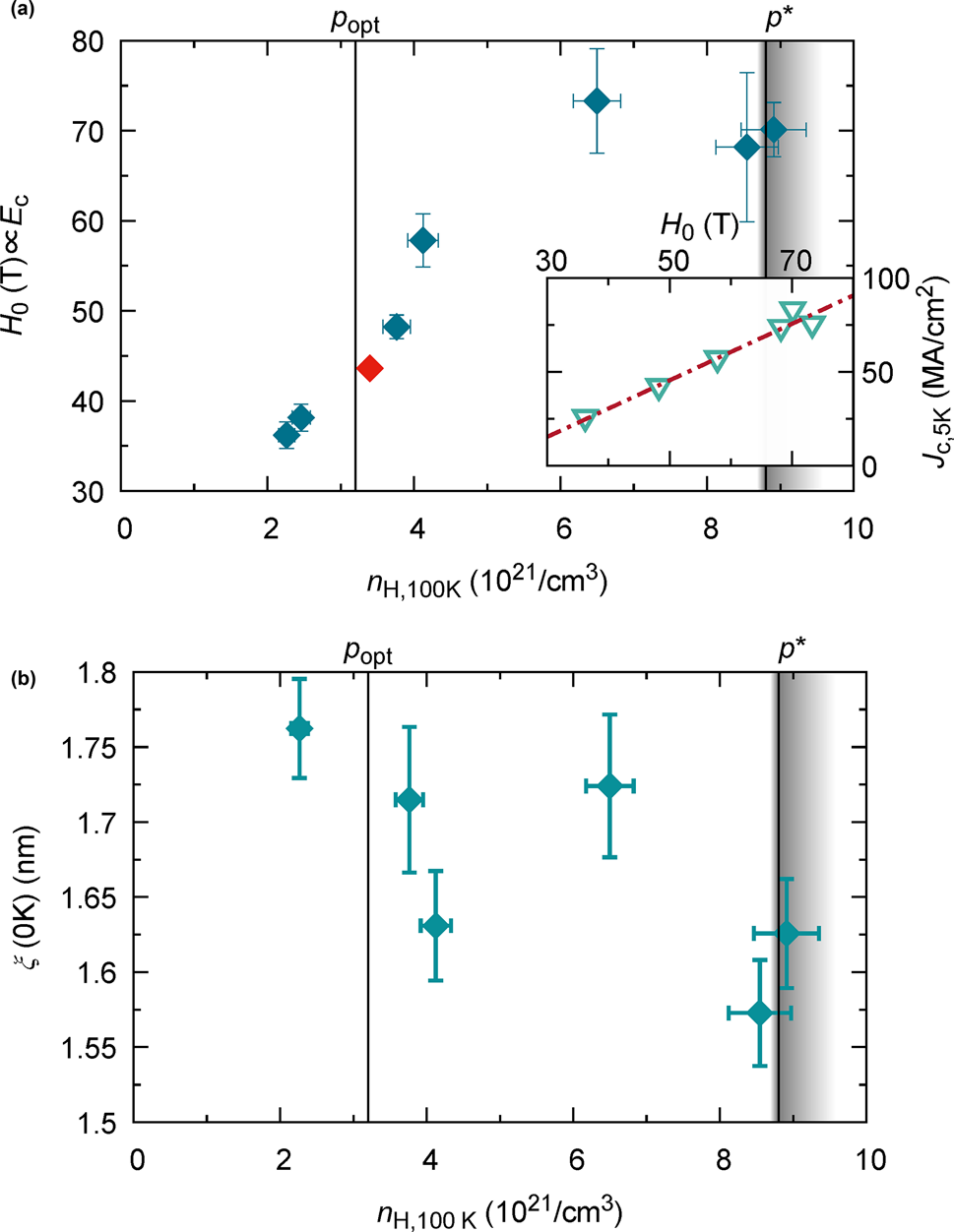
Charge carrier density dependence of superconducting parameters: (**a**) Characteristic magnetic field, ${H}_{0}$, versus charge carrier density at 100 K. ${H}_{0}$ is obtained by electrical measurements of the vortex glass transition line. It provides a measure of the pinning energy and thus is closely linked to the superconducting condensation energy, ${E}_{\mathrm{c}}$. The red diamond is reproduced from^23^ (280 nm, PLD), falling on the same line as our results. Inset shows ${J}_{c}\left(5\hspace{0.17em}\mathrm{K}\right)$ versus ${H}_{0}$, revealing a linear relation between these independently measured quantities. This emphasizes the strong correlation of the critical current density and the condensation energy. Dashed line is a guide to the eye. (**b**) Superconducting coherence length $\xi (0)$ as a function of Hall number at 100 K. $\xi (0)$ is obtained by electrical measurements of ${H}_{\mathrm{c}2}$, determined from flux flow resistivity analysis up to 9 T (SI-Fig. 5b). A weak decrease with increasing charge carrier doping is observed, which alone cannot account for the strongly enhanced ${J}_{\mathrm{c}}$ in the overdoped region. y-error bars correspond to uncertainties from the underlying fit procedures.

Vortex matter in HTS cuprates has demonstrated to be extremely rich with new vortex phases that were not expected from the low temperature superconductivity knowledge^46–48^, specially related to the high thermal energy and flux creep phenomena^49^ that these cuprates experience. In this context, the pinning energy (which is proportional to the condensation energy, ${E}_{c}$^41^) can be related to an effective activation energy determined from magnetoresistance measurements, which can be written generally as $U\left(H,T\right)={\left(1-\frac{T}{{T}_{c}}\right)}^{m}{\left(\frac{{H}_{0}}{H}\right)}^{\beta }$^50^, where $\beta$ is a constant close to unity and $m$ a material dependent parameter. The characteristic magnetic field ${H}_{0}$ is proportional to the pinning energy, and thus closely related to the condensation energy^23^. We have obtained ${H}_{0}$ by analysing the vortex glass transition line, given by the empirical formula ${H}_{\mathrm{G}}={H}_{0}{\left[\frac{1-t(H)}{t(H)}\right]}^{1/\beta }$^51–53^, with $t(H)={T}_{\mathrm{G}}(H)/{T}_{c}$, where ${T}_{c}$ is the critical temperature at zero field and ${T}_{\mathrm{G}}(H)$ the field dependent vortex-glass transition temperature. We find that ${H}_{0}$ depends on the Hall number, as shown in Fig. 5a. As ${n}_{\mathrm{H}}$ increases from $2$ to $9\cdot {10}^{21}$/cm^3^, ${H}_{0}$ doubles from $35$ to $>70$ T. Thus, suggesting an increase of the condensation energy,${E}_{\mathrm{c}}$, of the order of a factor two within this doping range. This is in agreement with the trend observed from the analysis of the condensation energy, ${E}_{\mathrm{c}}$, determined from measurements of the specific heat jump in Bi2212^54^ and YBCO^55^, and the upper and lower critical fields, ${H}_{\mathrm{c}1}$ and ${H}_{\mathrm{c}2}$ in YBCO^17^, which can be correlated, according to BCS theory, to the zero temperature condensation energy per unit volume, ${E}_{c}\propto \Delta C$ and ${E}_{\mathrm{c}}\propto {H}_{\mathrm{c}}^{2}$. In these two cases, ${E}_{\mathrm{c}}$ also approximately doubles from optimal to the critical doping^17,56^. In the inset of Fig. 5a we report a linear dependence of ${J}_{c}(5\hspace{0.17em}\mathrm{K})$ on ${H}_{0}$, which reinforce the condensation energy,${E}_{c}$, as the underlying quantity governing both parameters, thus further suggesting ${{H}_{0}\propto E}_{c}$.

To verify the plausibility of our results, we analyse the expected relationship between the depairing current density, ${J}_{\mathrm{d}}$, the condensation energy, ${E}_{c}$ and the charge carrier density, ${n}_{\mathrm{H}}$. Therefore, we consider the condensation energy per pair $u(T)$, which is equal to the critical kinetic energy at zero temperature:

2 $$u\left(0\right)=\frac{1}{2}m{v}_{c}^{2},$$

with the depairing critical velocity of a pair ${v}_{c}$ and $m$ the mass of the electron pair. The condensation energy per pair is equal to the condensation energy per unit volume divided by the superconducting pair density ${n}_{s}$, hence $u\left(0\right)={E}_{c}(0)/{n}_{s}$. Using the critical velocity, we can generally write the depairing critical current density as

3 $${J}_{d}=e{(2n}_{s}){v}_{c}.$$

Combining Eq. (2) and (3) we obtain

4 $${J}_{d}^{2}\propto {n}_{s}{E}_{c}.$$

**Figure 6:**
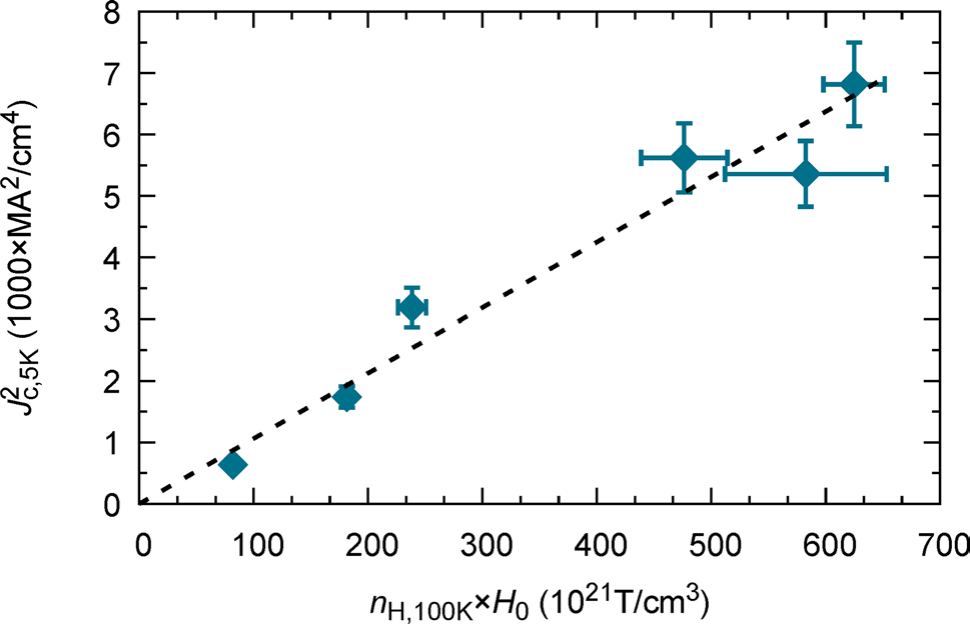
Experimental verification of Eq. (4), ${\mathrm{J}}_{\mathrm{c}}^{2}\propto {\mathrm{n}}_{\mathrm{H}}{\mathrm{E}}_{\mathrm{c}}$, using ${\mathrm{E}}_{\mathrm{c}}\propto {\mathrm{H}}_{0}$. The three parameters ${\mathrm{J}}_{\mathrm{c}}$, ${\mathrm{n}}_{\mathrm{H}}$ and ${\mathrm{E}}_{\mathrm{C}}$ are derived experimentally, in this work, from independent measurements.

Therefore, we expect that the square of the depairing critical current density varies as the superconducting pair density multiplied by the condensation energy. We further assume that ${n}_{s}$ varies with the measured Hall carrier density ${n}_{\mathrm{H}}\propto 2{n}_{s}$. Therefore, we expect that going from optimal doping to the highest achieved over-doping, ${n}_{H}$ increases by a factor of 3, while ${H}_{0}$ increases by a factor of 2. From the derived expression, the depairing critical current density is expected to increase over that range by a factor of 2.5, which is consistent with our results reported in Fig. 4a, suggesting that the measured self-field inductive critical current density varies similarly to the depairing critical current density. Equation 4 is experimentally verified in Fig. 6 with ${J}_{c}$, ${H}_{0}$ and ${n}_{\mathrm{H}}$ experimentally determined values. Thus, we believe that the increase of ${J}_{c}$ with ultrahigh critical currents up to p* is fully consistent with an increase of the condensation energy, ${E}_{c}$, in YBCO films, in agreement with previous results determined from specific heat^56^ and critical fields^17^ measurements. Some of these works have suggested the existence of a peak in the condensation energy ${E}_{\mathrm{c}}$, and thus in the density of states, ${N}_{\mathrm{F}}$, at p* when the PG closes and cuprates enter the strange metal state before reaching the Fermi liquid behaviour^15,17,27,56^. This is one of the features of a QCP irrespective of the details of the microscopic model used to describe the PG formation^23,27,57,58^. Unfortunately, this has been measured up to now only in Ca-doped YBCO and La_1.8-x_Eu_0.2_Sr_x_CuO_4_^27^ and iron-pnictide superconductors^59–62^, but it could suggest a universal behaviour. It would be very interesting to be able to overdope these YBCO films beyond the present p* value to confirm also the peak in ${E}_{\mathrm{c}}$.

In this work, we have evaluated the self-field inductive ${J}_{c}$ from saturated SQUID magnetization curves, to ensure being in the vortex pinning regime^63,64^, which is further confirmed by the strong increase of ${J}_{c}\, \mathrm{ with }\,{n}_{s}$ also for 7 T (Fig. 4b). Hence, variations of the coherence length might contribute to the increase of the critical current, as the pinning energy, ${U}_{p}(0)={E}_{c}(0){\xi (0)}^{3}$, scales with $\xi {\left(0\right)}^{3}$^56^ and the coherence length itself exhibits a dependence on doping^65–67^. To be able to exclude that a major contribution arises from changes in $\xi \left(0\right)$, we have analysed $\xi \left(0\right)$ for several samples spanning a broad doping range, as shown in Fig. 5b. $\xi \left(0\right)$ is extracted from electrical measurements of the flux flow resistivity up to 9 T as described in^68–70^. We observe only a small variation of $\xi (0)$, while the Hall number changes by more than a factor 4. Thus, the ${J}_{c}$ enhancement due to this modified coherence length would account for an upper limit of 25%, much less than the observed increase of > 300% within the doping range from $2$ to $8\cdot {10}^{21}$/cm^3^. Thus, we conclude that the enhancement of ${J}_{c}$ observed is mainly governed by the modified condensation energy due to the increase of oxygen doping.

### Consequences in pinning behaviour and comparison with nanoengineered films

**Figure 7:**
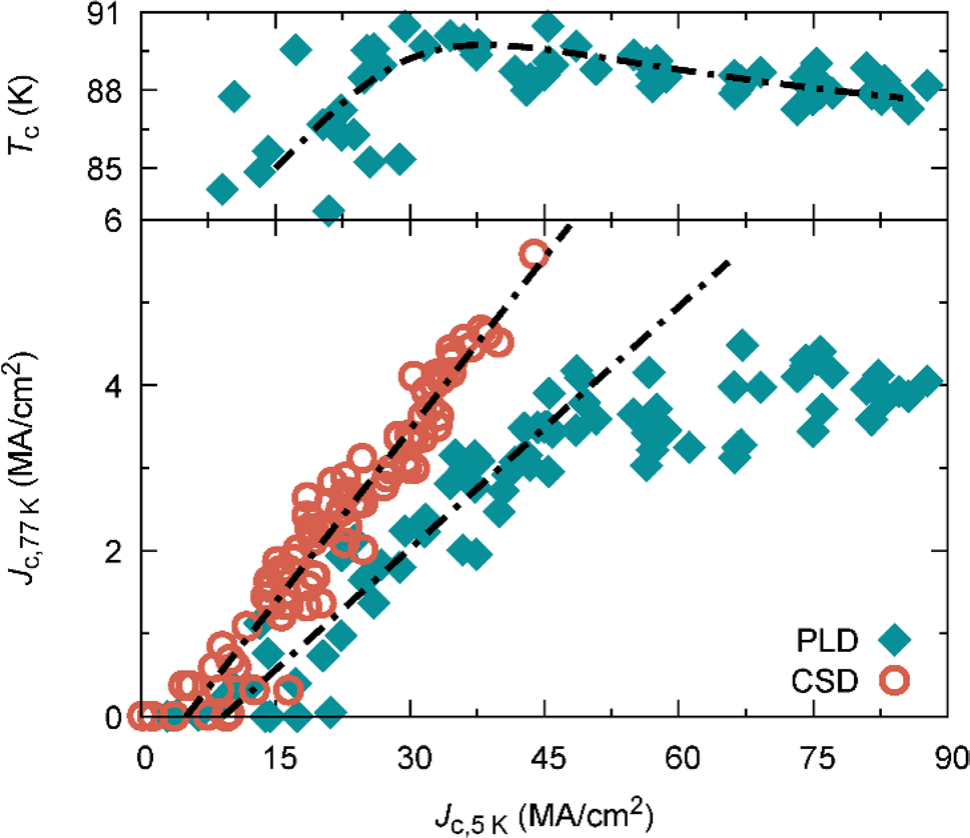
Superconducting physical properties correlations for PLD and CSD films. ${T}_{c}$ dependence with self-field inductive ${J}_{c}(5\hspace{0.17em}\mathrm{K})$ (upper panel) showing the intrinsic relation of both quantities with the charge carrier density. Self-field ${J}_{c}(77\hspace{0.17em}\mathrm{K})$ as a function of self-field ${J}_{c}\left(5\hspace{0.17em}\mathrm{K}\right)$ for YBCO films obtained by PLD and CSD (lower panel). The different linear trends for ${J}_{c}(5\hspace{0.17em}\mathrm{K})$<50 MA/cm^2^ reveal growth dependent pinning defect landscapes, with possibly higher strong-pinning contribution in CSD films. With increasing ${J}_{c}(5\hspace{0.17em}\mathrm{K})$ in PLD films, ${J}_{c}(77\hspace{0.17em}\mathrm{K})$ saturates, probably due to a reduced contribution of weak pinning at high temperatures and the proximity to the superconducting transition in the overdoped regime, as indicated in the upper panel by a decreased ${T}_{c}$ for high ${J}_{c}(5\hspace{0.17em}\mathrm{K})$ films. All lines are guide to the eye.

**Figure 8:**
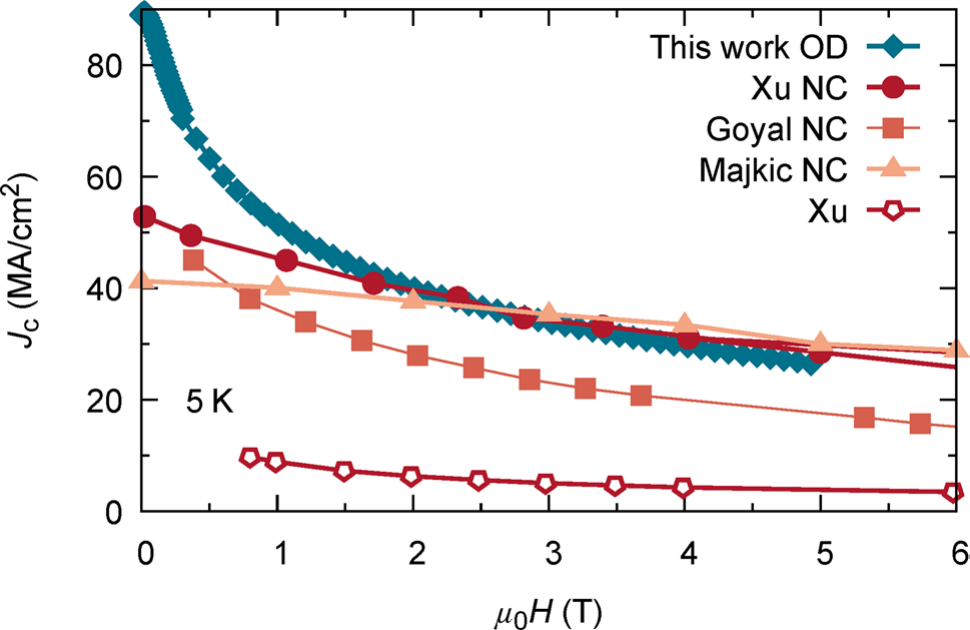
${J}_{c}(H\parallel c)$ at 5 K of some best performing YBCO films. Field dependence of reported high critical current densities for several nanocomposite (NC) YBCO thin films in comparison with overdoped (OD) YBCO obtained in this work (PLD). Overdoped YBCO exhibits a remarkable self-field inductive ${J}_{c}$, almost 60% higher than previous record films, compensating the fast decrease of ${J}_{c}(H)$ at low fields as typical for pristine YBCO films. From literature reproduced results cover the currently best-practice strategies of nanoengineering YBCO of coated conductors: 15% Zr doped (Gd,Y)BCO (Xu NC, MOCVD, at 4.2 K, reproduced under CC-BY)^42^, nanoscale defected REBCO with 4% BZO (Goyal NC, PLD, at 5 K, reproduced under CC-BY)^78^, REBCO with 15% Zr addition (Majkic NC, MOCVD, at 4.2 K, raw data was kindly provided by the author)^79^. Additionally we show a pristine YBCO film (Xu, MOCDV, at 4.2 K, reproduced with permission from AIP publishing) as reported in^72^.

YBCO films are very sensitive to atomic and nanoscale defects due to the small coherence length in high temperature superconductors, which in addition leads to strong thermal fluctuations, especially at high temperatures, which strongly hinder vortex pinning. The consequence is that very different behaviour is observed for different pinning centres at different temperatures. While columnar defects are more advantageous at high temperatures, weak uncorrelated pinning sites strongly contribute to the overall pinning force at reduced temperatures^41,71^. The effect of random point defects is observed to be more significant at low temperatures^10^. ${J}_{c}(77\hspace{0.17em}\mathrm{K})$ vs ${J}_{c}(5\hspace{0.17em}\mathrm{K})$ is shown in Fig. 7 for films grown by PLD and CSD. In both types of samples, we observe a strong correlation between ${J}_{c}$ at the two different temperatures up to about ${J}_{c}\left(5\hspace{0.17em}\mathrm{K}\right)=50$ MA/cm^2^. However, CSD and PLD films follow different trends, which may be attributed to different pinning efficiencies associated to two different microstructures. CSD films typically preserve a much stronger distorted matrix, giving raise to strong pinning in strained regions, being more efficient at higher temperatures^12^. Above ${J}_{c}\left(5\hspace{0.17em}\mathrm{K}\right)=50$ MA/cm^2^, PLD films show a deviation from the initial linear relation with ${J}_{c}(77\hspace{0.17em}\mathrm{K})$, resulting in a much weaker dependence (${J}_{c}\left(77\mathrm{K}\right)$ increases from 3 to 4 MA/cm^2^, while ${J}_{c}\left(5\hspace{0.17em}\mathrm{K}\right)$ almost doubles from 50 to 90 MA/cm^2^). We propose that this saturation is caused by the reduced efficiency of weak pinning sites at high temperatures. Notice, in Fig. 7 upper panel, the consistency of ${T}_{c}$ with self-field ${J}_{c}(5\hspace{0.17em}\mathrm{K})$, further demonstrating the intrinsic relationship of these two magnitudes with the charge carrier density (Figs. 7, 8).

The strong increase of ${J}_{c}$ with doping motivates for a comparison of the in-field ${J}_{c} (H)$ properties with reported values from other optimized approaches, including the more recent nanoengineering of the microstructure by the embedding of nanoparticles and nanorods in HTS films and coated conductors. ${J}_{c}(H\parallel c)$ at low temperatures is shown in Fig. 8 for various different samples (see Figure caption for details on sample composition). The most striking feature is the strong enhancement of the inductive ${J}_{c}$ at self-field (${\mu }_{0}H=0$) in the overdoped film, compared to conventional approaches by nanodefecting the matrix, as discussed above. Up to ${\mu }_{0}H=2$ T, overdoping enables an outstanding increase of ${J}_{c}(H)$. Weak pinning, the expected main contribution in pristine YBCO films, is known to rapidly decrease with small magnetic fields^10^, explaining the rapid decrease of ${J}_{c}(H)$ below 1 T for the overdoped PLD sample. However, the extraordinary high self-field inductive ${J}_{c}$ asserts high ${J}_{c}(H)$ at even intermediate fields, comparable to the best performing nanocomposites, whose pinning is governed by 1D nanorods and strained regions around 3D nanoparticles. We also show a representative example of a pristine optimally doped YBCO film, as reported in^72^, highlighting the potential of overdoping YBCO. We notice that in this study, ${J}_{c}$ was obtained by magnetisation measurements, so these results should better be compared with other inductive measurements, but we want to stress that typically, inductive measurements do suffer from extensible flux-creep effects specially at high magnetic field. Therefore, we encourage future work achieving the overdoped state in nanoengineered films to reach ultrahigh critical current densities also at high magnetic fields.

## Discussions

We have fabricated overdoped YBCO thin films by means of different post-processing oxygen heat treatments being able to tune the doping state and reach the critical doping value p* = 0.19, close to the Quantum Critical Point. The overdoped state is confirmed by a small decrease of ${T}_{c}$ and a transition of the normal state charge carrier density from $n=p$ to $n=1+p$, where *p* is the doping state of the Cu_2_O planes, in agreement with the proposed reconstruction of the Fermi surface above optimal doping (*p* = 0.16). However, this result is remarkable, as $n$ was obtained above the onset of superconductivity at 100 K, preserving the expected behaviour from the limit $T\to 0$. We suggest that low temperature measurements at high fields, necessary to suppress superconductivity, would be highly interesting to reveal the further temperature evolution of ${n}_{\mathrm{H}}(T)$ in these thin films.

The evaluated overdoped regime is characterised by an increase of the condensation energy, leading to extraordinary self-field ${J}_{c}$ values at low temperatures, up to one fifth of the depairing current, reaching 90 MA/cm^2^ at 5 K. We experimentally demonstrate a strong increase of ${J}_{c}$ with the charge carrier density, ${\mathrm{n}}_{\mathrm{H}}$, at self-field and high magnetic fields of 7 T up to the critical point p*, and we verified the theoretical relation between ${J}_{c}$, ${E}_{c}$ and ${\mathrm{n}}_{\mathrm{H}}$ (Eq. 4) from three independent experimental parameters. The distinct behaviour of ${J}_{c}$ observed at 5 K and 77 K, suggests that we have been able to modify the weak pinning individual strength through the modification of the condensation energy by doping. *p*-doping strategies with oxygen post-processing treatments are expected to be scalable and uniform in long length, therefore, we envisage a viable hybridisation of overdoping and nanoengineering of YBCO films, which offers powerful prospects to further push prevailing limitations of dissipation-free current transport in cuprate superconductors and Coated Conductors at high magnetic fields of interest for applications. We reinforce the interest to find ways to overdope YBCO films beyond the present critical value p* to confirm the existence of a peak in condensation energy, ${E}_{c}$, at higher doping levels and shine light on the consequences of crossing the Quantum Critical Point.

## Methods

### Film fabrication

The YBa_2_Cu_3_O_7-δ_ thin films are grown using chemical solution deposition (CSD) and pulsed laser deposition (PLD) on 5 × 5 mm^2^ LaAlO_3_ (100) and SrTi O_3_ (100) single crystal substrates with thicknesses of 200 nm (PLD) and 250 nm (CSD), respectively. In case of CSD, the stoichiometric amount of precursor metal trifluoroacetate salts is dissolved in an alcoholic solution and deposited by spin-coating, followed by a pyrolysis (∼300 °C) and growth (∼800 °C) temperature treatment at *P*_*O2*_ = 0.2 mbar^73,74^. PLD layers are deposited at 800 °C at *P*_*O2*_ = 0.3 mbar with a pulse frequency of 5 Hz^75^. The PLD-targets were fabricated by Oxolutia SL (Spain) and consist of pressed and sintered, stoichiometric YBCO powder at 87% density. After growth, a 100 nm thick surface decoration layer of patterned Ag is deposited on the surface by sputtering at RT (SI-Fig. 1a). This Ag layer catalytically enhances oxygen exchange activity of YBCO during the following oxygenation process, as a job sharing mechanism facilitates the dissociation and incorporation of O_2_ (SI-Fig. 1b)^28,76^. Additionally, the silver coating provides good electrical contact for electrical measurements. Dewetting of the Ag layer into small islands with diameters of about 0.1 µm is observed at around 300 °C. However, this effect is not detrimental to its catalytic activity nor electrode functionality. Hole doping of YBCO is achieved by oxygen incorporation during the post growth oxygenation at 1 bar with an oxygen flux density of 0.16 l min^-1^ cm^-2^ at different intermediate temperatures (280–550 °C) with dwell times between 30 and 240 min.

### Structural characterisation

Layers obtained by either growth technique are epitaxial textured, twinned and highly c-axis oriented with no trace of secondary phases, as determined by X-ray diffraction measurements (Bruker D8 Discover), as shown in SI-Fig. 3a. In the presented films with thicknesses above 200 nm, no macroscopic strain due to lattice mismatch with the used substrates (LaAlO_3_ and SrTiO_3_) was observed.

The *c*-lattice parameter is obtained by HR-XRD measurements using the Nelson–Riley method, which allows the determination of the lattice parameter with very high precision, as aberration errors are minimised at very high angles (see SI-Fig. 3b). For optimally and overdoped films, we determined the doping number via the *c*-parameter, using the empirical equation $p={c}_{1}y+{c}_{2}{y}^{6}+{p}_{0}$ with $y=1-c/{c}_{0}$^20,77^. The prefactors ${c}_{i}$ depend on the sample type and growth process. In this work we have used values reported in^77^ (${c}_{0}=11.8447$, ${c}_{1}=11.491$, ${c}_{2}=5.17\cdot {10}^{9}$). A small systematic constant offset was corrected by introducing the additional parameter ${p}_{0}=-0.02$.

### Magnetic and electrical analysis

SQUID magnetometry (Quantum Design) was used to determine critical current densities via the width of the magnetisation loop, as explained in SI-Fig. 4a, using the Bean critical state model for thin discs.

In-depth electrical analysis was performed using a Physical Property Measurement System (Quantum Design) over a broad temperature range. Contacts for electrical measurements were glued with silver paint on top of 400 µm squared Ag electrodes sputtered at the corners of the films. Electrical measurements were performed in Van der Pauw and Hall configuration in fields up to 9 T $(H\parallel c)$, averaged over two permutations of the electrical contacts and positive and negative excitation current in DC mode. The studied YBCO films are highly twinned, therefore the influence of metallic CuO chains short circuiting the Hall voltage along the *b*-direction is neglected in the calculation of the Hall coefficient ${R}_{\mathrm{H}}$. The critical temperature at zero field, ${T}_{c}$, and the field dependent vortex-glass transition temperature, ${T}_{\mathrm{G}}(H)$, are determined from $\rho (T,H)$ measurements to the point where the electrical resistance in response to a small excitation current vanishes. The vortex-glass transition line ${H}_{G}={H}_{0}{\left[\frac{1-t(H)}{t(H)}\right]}^{1/\beta }$ is used to obtain the characteristic magnetic field, ${H}_{0}$, by linear fitting as explained in SI-Fig. 5a. From the same measurements, we were able to determine the upper critical field, ${H}_{c2}(T)$, and in the zero temperature limit, ${H}_{c2}(0)$, using the classical Werthamer-Helfand-Hohenberg relation, which in turn defines the coherence length, $\xi \left(0\right)$. The determination of ${H}_{c2}(T)$ is shown in SI-Fig. 5b.

## Supplementary Information

Supplementary Information

## Data Availability

The data that support the findings of this study are available from the corresponding authors on reasonable request.
