# Supplementary material for: Ultra-high critical current densities of superconducting YBa2Cu3O7-δ thin films in the overdoped state
Source: Sci Rep. 2021 Apr 14;11:8176. doi: 10.1038/s41598-021-87639-4 (PMC8047038; doi:10.1038/s41598-021-87639-4)
Supplement: Supplementary file 1 — Supplementary Information [file 41598_2021_87639_MOESM1_ESM.pdf]

Supplementary Information

Ultra-high critical current densities of superconducting $YBa_2Cu_3O_{7-\delta}$ thin films in the overdoped state

A. Stangl[1,*], A. Palau[1], G. Deutscher[2], X. Obradors[1], T. Puig[1,*]

[1]Institut de Ciència de Materials de Barcelona (ICMAB-CSIC) Campus de Bellaterra, 08193 Bellaterra, Barcelona, Spain

[2]Department of Physics and Astronomy, Tel Aviv University, 69978 Tel Aviv, Israel

* corresponding authors: teresa.puig@icmab.es, alexander.stangl@grenoble-inp.fr

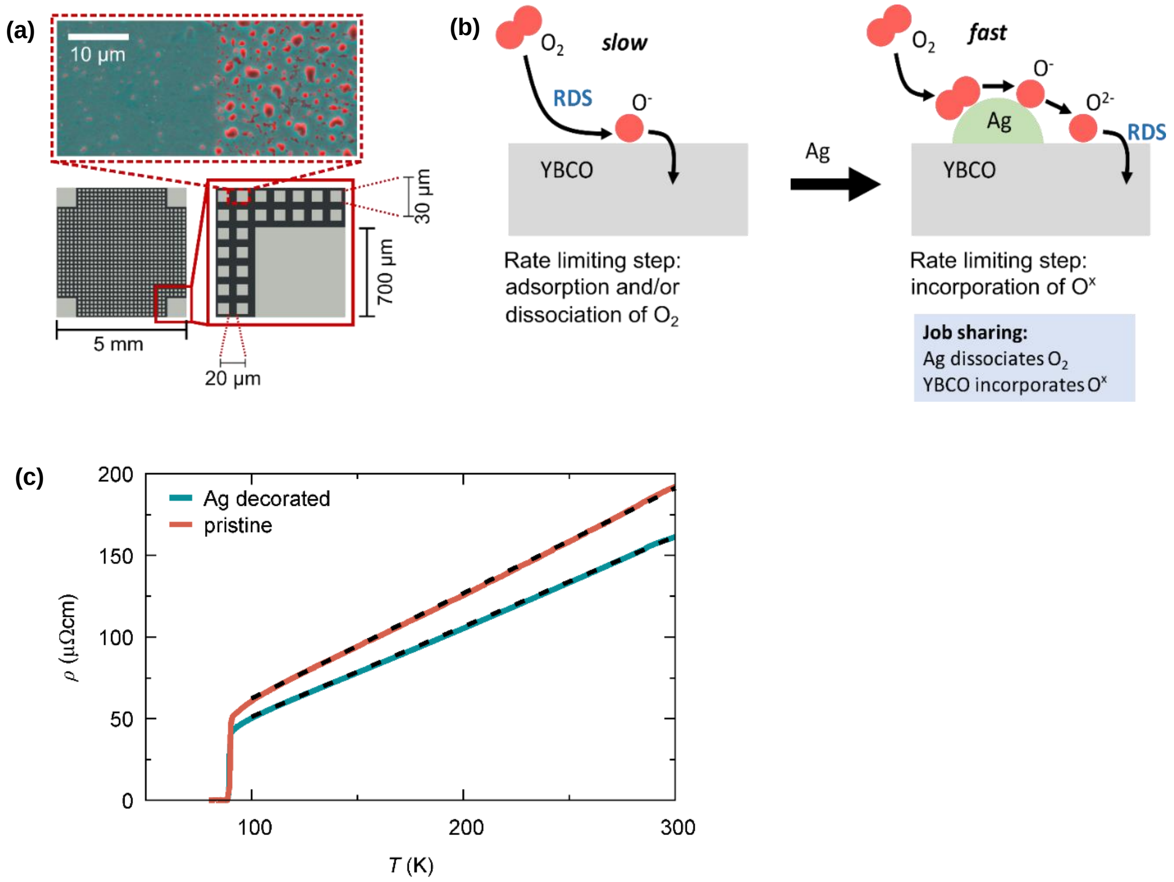

*SI-Figure 1: **Role of Ag in post oxygen heat treatment:** (a) Schematic of silver surface decoration using a 100 nm thick mesh of 30x30 µm squares on top of the superconducting film*

surface and 700 µm big pads in the corners used as electrodes for electrical measurements (as-deposited Ag surface coverage: 40 %). False colour SEM image in combined SE (secondary electron) and BSE (back-scattered electron) mode in the top panel shows dewetting of continuous layer into Ag islands as observed after heat treatments above 300 °C, reducing the coverage to about 10 %. (b) Role of Ag during the oxygen incorporation process. In pristine YBCO thin films oxygen exchange is rate limited (rate determining step, RDS) by adsorption and/or dissociation of molecular oxygen onto the film surface, resulting in a sluggish overall oxygen reduction reaction (ORR). Catalytic activity of Ag strongly accelerates these processes and oxygen is efficiently incorporated into the YBCO bulk at the triple phase boundary around the Ag particles even at low temperatures, enabling low temperature oxygen post treatments[1]. (c) Electrical resistivity measurements of Ag decorated and pristine YBCO 200 nm thick films, showing that Ag surface decoration layer does not affect electrical measurements. The small difference in $\rho(T)$ *is caused by different charge carrier densities, with the Ag decorated one having a* $n_H(100\,K)$ *of* 7.4×10^{21}*/cm³ in comparison to* 5.7×10^{21}*/cm³ in the pristine film.*

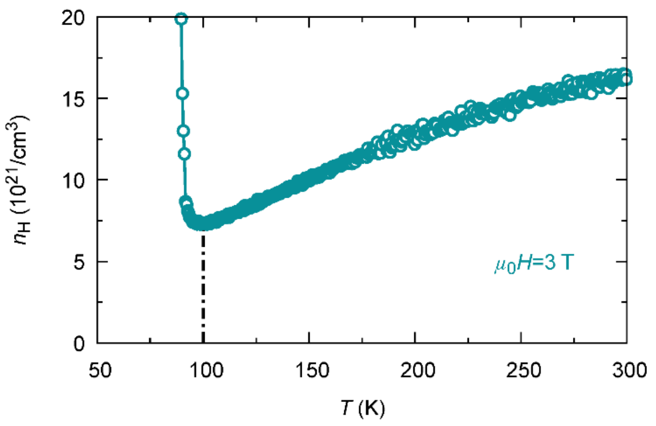

SI-Figure 2: ***Temperature dependent charge carrier density:*** *From Hall effect measurements at constant field (e.g.* $\mu_0 H = 3$ *T), the single band charge carrier density of YBCO was obtained via* $n_H(T) = \frac{1}{eR_H(T)}$ *as a function of temperature. Typically in YBCO single crystals, an anisotropy factor is introduced in the determination of the charge carrier density via the Hall constant, accounting for the contribution of the CuO-chains short-circuiting the Hall voltage along the b-direction. However, as here studied films are highly twinned and we do not observe in-plane*

anisotropy in our Hall measurements, we can safely neglect the effect of the metallic CuO-chains in the calculation of the charge carrier density.

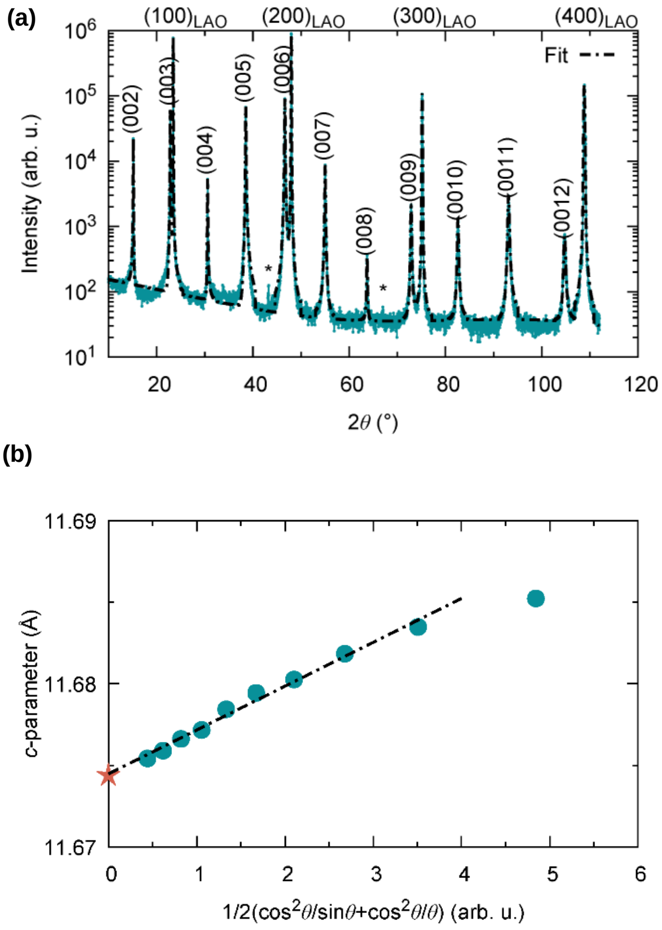

SI-Figure 3: ***c-parameter evaluation via HR-XRD:*** *(a) X-ray diffraction pattern of YBCO thin film (200 nm) on top of LAO (100) single crystal substrate. Substrate and film peaks can be very well reproduced using Voigt profiles. The background is substracted using a third order polynomial over the full range. Higher order reflection of the substrate peaks (h00) are marked with *. (b) Film c-parameter is obtained by extrapolation of* $c = \lambda/2\,sin\,\theta$ *to the intersect* $2\theta = 180\,°$ *via a linear fit using the Nelson – Riley formula (*c_{Film} *= 11.674 Å, as indicated with the red star). This method allows the determination of the lattice parameter with very high precision, since aberration errors are minimised at very high angles.*

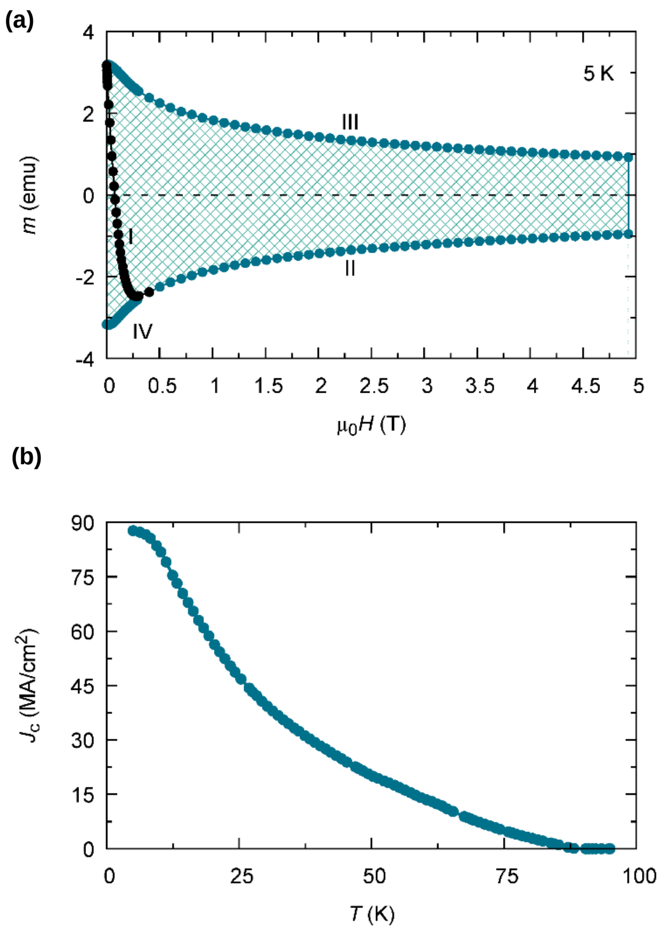

SI-Figure 4: ***Critical current density by SQUID remanent magnetisation measurements:*** *(a) Irreversible magnetic moment in the saturated $m - H$ hysteresis loop (blue dots) at 5 K. The area between the two branches, corresponding to increasing and decreasing magnetic field, is proportional to the critical current density, $J_c(H)$, through the Bean critical state model. The $m(H)$ hysteresis loop is determined starting from the initial magnetic moment branch (I), by increasing the field up to 5 T (II), subsequent decreasing to -5 T (III), followed by an increase (IV) to 0.3 mT to close the fully saturated loop. (b) Critical current as a function of temperature as obtained by SQUID measurements using Bean critical state model for a thin disc[2]: $J_c = 3\Delta m/2(r^3\pi t)$, considering an average radius $r = \sqrt{a^2/\pi}$ of the squared samples with side length a, the full width, Δm, of the saturated hysteretic cycle and the film thickness t.*

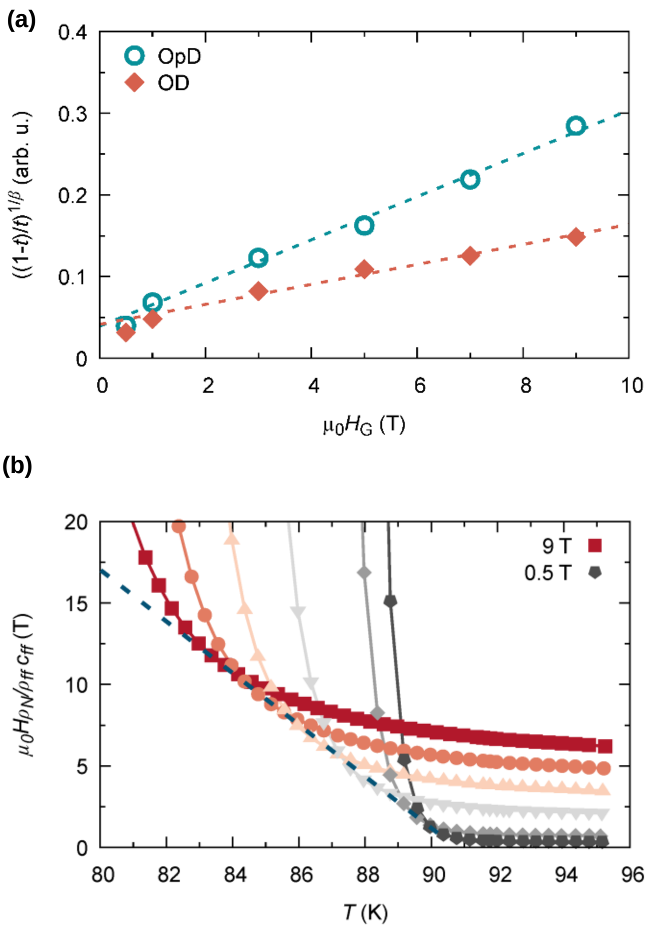

SI-Figure 5: ***Analysis of the pinning energy by electrical measurements:*** *(a) Evaluation of the vortex glass transition* $H_G = H_0 \left[\frac{1-t(H)}{t(H)}\right]^{1/\beta}$ *used to extract the characteristic magnetic field,* H_0*, which corresponds to the slope in the figure.* H_0 *values were determined from the linear fitting (dashed lines) as shown for an optimally and highly overdoped YBCO thin film, giving* $\mu_0 H_0 = 37\ T$ *and* $\mu_0 H_0 = 75\ T$*, respectively.* $t = \frac{T_0(H)}{T_c}$ *is the reduced zero-resistance temperature. The critical temperature at zero field,* T_c*, and the field dependent vortex-glass transition temperature,* $T_G(H)$*, are determined from* $\rho(T, H)$ *measurements (in Van-der-Pauw configuration) to the point where the electrical resistance in response to a small excitation current vanishes.* $\beta = 0.98$ *is a model parameter as reported in literature*[3]*. (b) The upper critical field,* $H_{c2}(T)$*, close to* T_c *is determined by the envelope (dashed line) of* $\mu_0 H \rho_N(T)/\rho_{ff}(H,T)c_{ff}$ *curves, measured at different magnetic fields. The* H_{c2} *zero temperature limit,* $H_{c2}(0)$*, is calculated using the classical Werthamer-Helfand-Hohenberg relation* $H_{c2}(0) = -0.69\, T_c \frac{\partial H_{c2}}{\partial T}|_{T_c}$*. The coherence length,* $\xi(0)$*, is then given by* $\mu_0 H_{c2}(0) = \phi_0/2\pi\xi(0)^2$*. The linear normal-state resistivity,* $\rho_N(T)$*, is determined*

above 100 K in zero field and extrapolated to low temperatures, $\rho_{ff}(H,T)$ *is the flux flow resistivity and* $c_{ff} = 1.45$ *is a constant* [4]*.*

1. Stangl, A. PhD Thesis on Oxygen kinetics and charge doping for high critical current YBCO films. (Universitat Autònoma de Barcelona, 2019). ISBN: 9788449087103. URL: http://hdl.handle.net/10803/667212.

2. Chen, D. X. & Goldfarb, R. B. Kim model for magnetization of type-II superconductors. *J. Appl. Phys.* **66**, 2489–2500 (1989).

3. Naqib, S. H. & Islam, R. S. Possible quantum critical behavior revealed by the critical current density of hole doped high-T c cuprates in comparison to heavy fermion superconductors. *Sci. Rep.* **9**, 1–8 (2019).

4. Bartolomé, E. *et al.* Intrinsic anisotropy versus effective pinning anisotropy in YBa2Cu3O7 thin films and nanocomposites. *Phys. Rev. B* **100**, 054502 (2019).
